# Long Medical Society

**Published:** 1886-04

**Authors:** H. P. Luckett

**Affiliations:** Sec’y.


### LONG MEDICAL SOCIETY.

F. E. DANIEL, M. D.

*Dear Doctor:*

AT the last regular meeting of Long Medical Society of this county a resolution was passed that the secretary furnish your JOURNAL the minutes, and essay that was read by Dr. Vermillion, of McDade. Yours respectfully,

H. P. LUCKETT, Sec'y.

February 12, 1886.—Long Medical Society, of Bastrop county, met in regular session, Dr. Robinson in the chair. Present: Drs. Hill, Garwood, Vermillion, Powell and Lucket. Essay, by Dr. Vermillion, as retiring President; also an interesting case reported by Dr. Powell.

H. P. LUCKETT, Sec'y.

